# FELIX: an algorithm for indexing multiple crystallites in X-ray free-electron laser snapshot diffraction images

**DOI:** 10.1107/S1600576717007506

**Published:** 2017-07-07

**Authors:** Kenneth R. Beyerlein, Thomas A. White, Oleksandr Yefanov, Cornelius Gati, Ivan G. Kazantsev, Nicolai Fog-Gade Nielsen, Peter M. Larsen, Henry N. Chapman, Søren Schmidt

**Affiliations:** a Center for Free-Electron Laser Science, DESY, Notkestrasse 85, 22607 Hamburg, Germany; b Institute of Computational Mathematics and Mathematical Geophysics, Lavrentieva 6, 630090 Novosibirsk, Russian Federation; c Department of Physics, Technical University of Denmark, DK-2800, Denmark

**Keywords:** serial crystallography, SFX, materials science, structural biology

## Abstract

A novel algorithm for indexing multiple crystals in snapshot X-ray diffraction images, especially suited for serial crystallography data, is presented. The algorithm, FELIX, utilizes a generalized parametrization of the Rodrigues–Frank space, in which all crystal systems can be represented without singularities. The new algorithm is shown to be capable of indexing more than ten crystals per image in simulations of cubic, tetragonal and monoclinic crystal diffraction patterns. It is also used to index an experimental serial crystallography dataset from lysozyme microcrystals. The increased number of indexed crystals is shown to result in a better signal-to-noise ratio, and fewer images are needed to achieve the same data quality as when indexing one crystal per image. The relative orientations between the multiple crystals indexed in an image show a slight tendency of the lysozme microcrystals to adhere on (10) facets.

## Abstract 2

The FELIX algorithm for indexing snaphot images containing multiple diffraction patterns is described and its performance is tested.

## 1. Introduction   

X-ray serial crystallography, SX, is a class of techniques that allows protein structure determination by merging intensities from snapshot diffraction patterns of many different microcrystals. The patterns can be collected using the short pulses of an X-ray free-electron laser (XFEL), called serial femto­second crystallography (SFX) (Chapman *et al.*, 2011 ▸), or using millisecond exposures at a microfocus synchrotron facility (Gati *et al.*, 2014 ▸). In most of these experiments the orientations and arrival times of crystals into the beam are random because of the necessity for fast sample replenishment (DePonte *et al.*, 2008 ▸; Hunter *et al.*, 2014 ▸; Sierra *et al.*, 2015 ▸; Stellato *et al.*, 2014 ▸; Weierstall *et al.*, 2014 ▸). The task of determining the number and orientations of the crystals in the recorded images is then left to the indexing algorithms. When the arrival of crystals is truly random, the number of diffraction patterns found in an image will follow Poisson statistics. Thus, the maximum fraction of one-crystal images is 36.8%, which is achieved when 63.2% of the images contain at least one pattern (hit fraction) (Park *et al.*, 2013 ▸). In this case, 27% will be multi-crystal images, with this fraction increasing with the hit fraction. Therefore, at some point, improving the time and sample consumption efficiency of serial crystallography experiments requires the ability to index multi-crystal images, even for non-interacting particles.

The intensities in multi-crystal images have been shown to carry useful information as long as spot overlap is low or properly treated. Spot overlap has been studied in a few high-resolution protein diffraction wedge datasets. This measurement consists of collecting a series of exposures while a large single crystal is continuously rotated. In one case, with four crystals of insulin simultaneously in the beam, spot overlap has been shown to affect less than 1% of the recorded reflections (Paithankar *et al.*, 2011 ▸). However, for six lattices of bovine pancreatic trypsin, 20% of the reflections were found to overlap, but mostly in the area away from the spot center (Gildea *et al.*, 2014 ▸). Less overlap can be expected for monochromatic snapshot multi-crystal images because a narrower slice of reciprocal space will lead to fewer spots on the detector.

The subtract-and-retry approach to multi-crystal indexing iteratively uses single-crystal indexing algorithms to find a dominant lattice in an image, subtract the associated spots and retry indexing. This approach has been shown to be effective to index up to six crystals when applied to wedge data (Powell *et al.*, 2013 ▸; Gildea *et al.*, 2014 ▸; Sauter & Poon, 2010 ▸). However, this presents a lesser challenge than the snapshot case, as the controlled rotation provides multiple views of the same group of crystals. It has also been applied in a few cases to XFEL snapshot data, but was only shown to index images containing two or three lattices (Hattne *et al.*, 2014 ▸; Ginn *et al.*, 2016 ▸).

An algorithm called *Grainspotter* (Schmidt, 2014 ▸), part of the *Fable* software platform (*Fable*, 2003 ▸), utilizes the properties of Rodrigues–Frank (RF) space to index wedge datasets for polycrystalline inorganic materials structure determination (Sørensen *et al.*, 2012 ▸). It has been used to index insulin and hen egg white lysozyme datasets collected at a synchrotron radiation facility with multiple crystals in the beam (Paithankar *et al.*, 2011 ▸). Related algorithms have also been used for small-molecule structural refinement from multi-crystal samples (Schmidt *et al.*, 2003 ▸; Vaughan *et al.*, 2004 ▸). A further application includes high-pressure science, where structural determination of individual (Mg,Fe)SiO_3_ post-perovskite crystals has been obtained in a diamond anvil cell (Zhang *et al.*, 2013 ▸).

Typically, when indexing multi-crystal data obtained from a rotation series, only a subset of diffraction spots on the detector are selected for the indexing procedure. This set is chosen to contain well separated *hkl* families to ensure unique assignment. Since rotation series cover a large volume of reciprocal space, there is sufficient information in the reduced data set for robust multi-crystal indexing. In contrast, for an SX diffraction snapshot all of the recorded diffraction spots arising from many *hkl* families are needed for RF space multi-crystal indexing. This is still a tractable problem when only a few tens of crystals are expected per image, but is not possible for the case of a polycrystalline material, where thousands of crystals in the beam require a rotation series to be indexed (see *e.g.* Wright, 2017 ▸; Sharma *et al.*, 2012 ▸; Schmidt, 2014 ▸).

Consequently, we created a new RF-space-based algorithm called FELIX for the scenario of snapshot images with patterns from crystals with closely positioned or overlapping *hkl* families. This indexer is implemented in a free and open-source program that has also been interfaced with the *CrystFEL* data analysis package (White *et al.*, 2012 ▸, 2016 ▸). In the following article, we begin by describing the workflow of the FELIX algorithm. Then, its ability to sort out overlapping *hkl* families is tested by indexing simulated multi-crystal images with patterns of different symmetries. Finally, the indexer is applied to experimental SX data collected from lysozyme microcrystals. The resulting structure and data statistics are compared with that obtained when only indexing one crystal per image. The article concludes with some discussion of foreseen future developments of the algorithm.

## 2. The FELIX algorithm   

**Figure 1:**
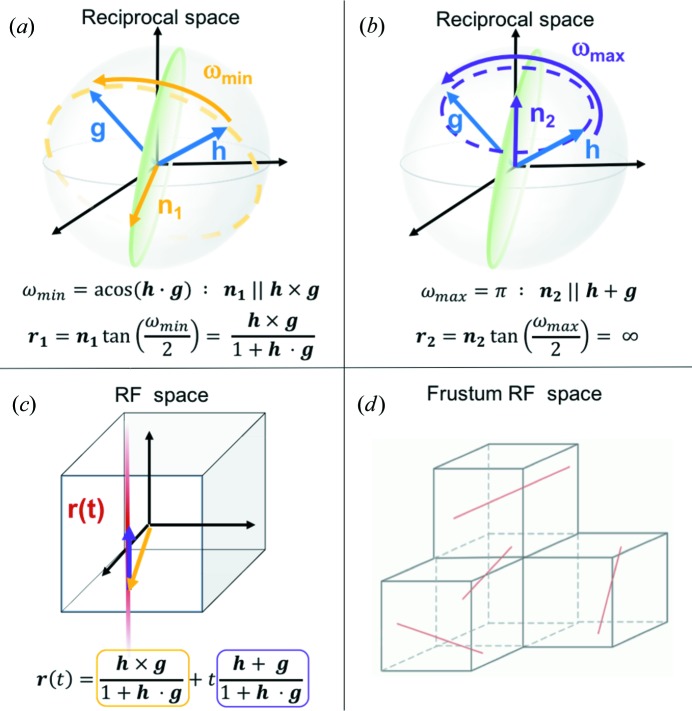
(*a*), (*b*) Limiting cases of the rotation operations that bring a Bragg spot **h** onto an observed scattering vector **g**. The rotation axis requiring the minimum rotation, 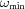, is parallel to **h** 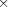 **g**, while that requiring the maximum rotation, 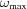, is parallel to **h** + **g**. All possible rotation axes must satisfy **h**  **n** = **g**  **n** and thus lie on the green circular plane that bisects the two vectors. (*c*) The full set of such rotations can be expressed as a linear combination of the two limiting cases, as given by the equation for **r**(*t*). From equation (1), these limiting cases map to vectors in RF space, and the expression for the geodesic, **r**(*t*), is an infinite straight line. (*d*) To avoid searching an infinite space, FELIX maps RF space into four frustums, shown as the four cubes in the image. All of the surfaces of the frustums are mathematically connected, so that a geodesic passing through one surface continues on in the neighboring frustum. The red lines shown in each frustum are then actually a single geodesic that is unbroken when the surface connectivity is applied.

The presence and position of a single Bragg spot on a detector strongly constrains the possible crystal orientations but does not allow for a unique solution. As illustrated in Fig. 1 ▸, this reduced set of orientations is given by the operations that bring a presumed Bragg reflection, **h**, onto the observed spot, **g**. This set defines a geodesic, which maps to a straight infinite line in RF space. The FELIX algorithm then searches the full RF space for intersections of the geodesics predicted from each spot and choice of *hkl* to solve for the orientation of a crystal. This is in contrast to the *Grainspotter* algorithm, which only searches sub-volumes of RF space and uses predominately the spots from well separated *hkl* families.

A crystallographic orientation 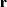 is represented as a vector in RF space defined by a rotation axis 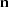, 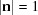, and angle ω (Morawiec & Field, 1996 ▸): 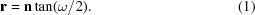
The divergence of the tangent function in this equation indicates that RF space is not Euclidean and has infinite size for rotations approaching 180°, making a direct search of this space intractable, especially for monoclinic and triclinic crystal systems. To overcome this problem, FELIX maps the full orientation space into four finite volumes called frustums. Each represent a different part of the orientation space. These frustums are illustrated in Fig. 1 ▸(*d*). Within each frustum the properties of RF space are retained, so geodesics still exist as straight lines and continue into neighboring frustums *via* connected boundary conditions. The mathematical details of geodesics in frustums are described in the manuscripts of Kazantsev *et al.* (2009 ▸) and Kazantsev & Schmidt (2014 ▸). FELIX segments each frustum into a user specified number of voxels (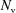) along each dimension of a frustum (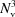 voxels in total) when searching for geodesic intersections.

**Figure 2:**
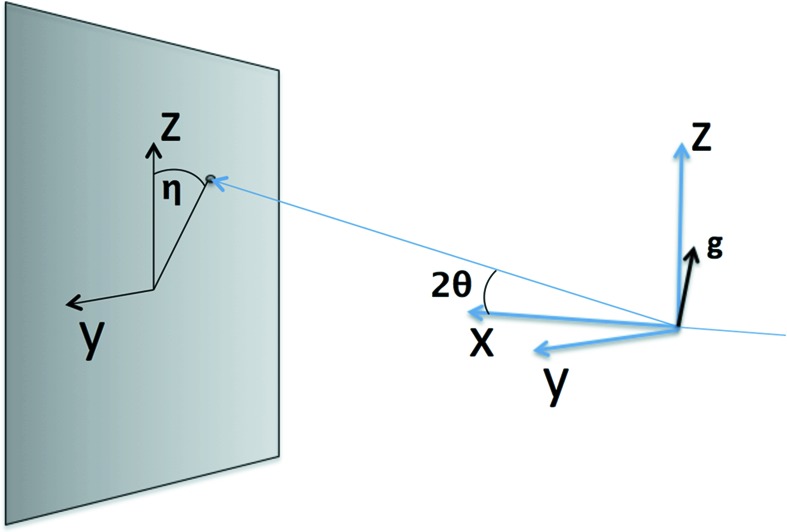
Sample–detector coordinate systems in FELIX. The sample is imagined to be at the center of the *xyz* axis, with the beam along the *x* axis. The sample then scatters radiation at angles 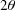 and η onto the detector, represented as a grey plane in the illustration. The corresponding **g** vector is shown at the origin.

As input, FELIX takes a list of observed spots on the detector that have been mapped into reciprocal space (**g** vectors), information on the crystal unit cell, and a set of cutoff parameters. The **g** vector is parametrized through the wavelength of the X-ray beam, λ, and the angles η and θ,
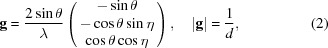
where *d* is the lattice spacing. A schematic view of how the **g** vector relates to the sample–detector coordinate system is shown in Fig. 2 ▸.

A list of *hkl* families and theoretical reciprocal space vectors, **h**, are either supplied or generated in FELIX from a specified unit cell and space group using the SgInfo library (Grosse-Kunstleve, 1994 ▸). A list of (**g**, **h**) vector candidate pairs is initially generated by comparing each **g** vector with the *hkl* families, 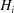, and accepting those for which 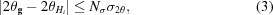
where 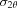 and 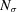 are user-defined estimates of the 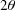 uncertainty and a scale factor, respectively. For each (**g**, **h**) candidate pair, a geodesic is propagated through the frustums *via* ray tracing, incrementing a counter in each voxel that it visits.

After processing all (**g**, **h**) candidates, FELIX searches for orientation candidates by identifying voxels corresponding to local maxima in the frustums. Each local maximum, *V*, that fulfills the following user-defined criterion is considered an orientation candidate: 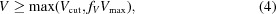
where 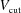 is the minimum number of required visits and 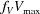 is a fraction parameter, 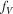, scaled by the most visits, 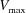. For each orientation candidate, the set of **g** vectors that are closest to the predicted lattice in reciprocal space are selected. A user-defined upper bound on the deviation between **g** and **h** is given by 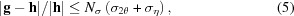
where 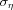 is a user-defined estimate of the uncertainty in η. Each point **h** can be associated with an equivalent rotation of the crystal around the *z* axis, 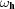. An upper bound on the equivalent rotation angle is used for the pre-selection of the (**g**, **h**) pairs considered in equation (5), given by another user-defined parameter, 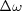: 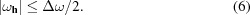
Finally, orientation fitting and outlier removal is performed using the same procedure as in *Grainspotter* (Schmidt, 2014 ▸). In order to accept an orientation, at least 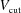 **g** vectors must remain after outlier removal. Also, the completeness of predicted spots that match the observed **g** vectors must be greater than a specified fraction (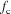). In addition, if the set of **g** vectors has a uniqueness fraction, *u*, that overlaps with an already accepted orientation, only the orientation with the most **g** vectors is kept.

As the symmetry of a crystal space group decreases from cubic to triclinic systems, the number of *hkl* families that can agree with a given **g** vector increases. This increases the number of overlapping *hkl* families and leads to more geodesics which must be traced. This causes a longer calculation time, as well as more opportunity for FELIX to return false positives. Therefore, in the following section we describe the results of simulations studying the accuracy of FELIX applied to different crystal systems.

## 3. Performance   

### 3.1. Simulated data   

Three simulation scenarios were chosen to match potential application areas for multi-crystal indexing in serial crystal data collection. The following three cases were studied: RHO-G6, one of the largest recently solved zeolite structures (Guo *et al.*, 2015 ▸); hen egg white lysozyme, a protein standard solved to high resolution *via* serial crystallography (Boutet, 2013 ▸); and AT
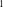
R, a G-protein coupled receptor structure recently solved by serial femtosecond X-ray diffraction (Zhang *et al.*, 2015 ▸). The crystal structures of these molecules have cubic, tetragonal and monoclinic lattice symmetries, respectively. In each case, images were simulated containing multiple overlaid crystal diffraction patterns, and the orientations determined by FELIX were compared with the known values. A list of unit-cell, crystal symmetry and simulated experimental parameters is given in Table 1 ▸.

**Figure 3:**
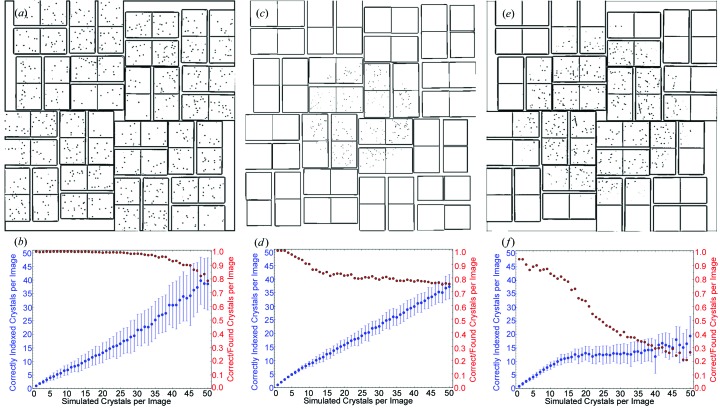
(*a*), (*c*), (*e*) Simulated images containing diffraction patterns from five crystals each of RHO-G6, lysozyme and AT
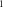
R, respectively. The spots in the images have been enlarged for the purposes of illustration. (*b*), (*d*), (*f*) Respective trends of the average number of correctly indexed crystals and fraction of correctly found crystals as the number of crystals in the simulated image is increased for the three aforementioned crystal systems. The error bars on the number of correctly indexed crystals depict the standard deviation of this quantity over a set of 100 independent simulations.

Diffraction patterns without background were simulated using the *CrystFEL* program *partial_sim*, which takes a list of Bragg intensities and places single-pixel-sized Bragg spots on a detector, considering a spherical model for partiality (White *et al.*, 2013 ▸). Bragg intensities were calculated from the published CIF and PDB files using the programs *iotbx.cif* of *cctbx* (Gildea *et al.*, 2011 ▸) and *SFALL* of *CCP4* (Agarwal, 1978 ▸), respectively. The images were simulated assuming the tiled CSPAD detector geometry of the Linac Coherent Light Source (LCLS) (Hart *et al.*, 2012 ▸; Philipp *et al.*, 2011 ▸). The resolution of each case was chosen to reflect experimentally realistic conditions. Example images containing five overlapping crystal patterns are shown in Fig. 3 ▸.

The simulated multi-crystal images were then indexed using the FELIX algorithm, called by the *CrystFEL* program *indexamajig*. Peaks were found using the zaef algorithm (Zaefferer, 2000 ▸). The indexing accuracy was accessed by comparing the obtained orientations with the known values considering crystal symmetry. For each crystal system, a set of images containing between one and ten patterns per image was simulated, and a matrix of FELIX parameters were tested to find those that maximized the overall indexing accuracy. The dominant crystal-symmetry-dependent parameters were found to be 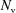, 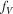, 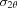 and 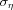. Both the speed and the accuracy of the algorithm were sensitive to these parameters. The best values for each case are listed in Table 1 ▸.

Using these best parameters, the trends shown in Fig. 3 ▸ for the fraction of accurately indexed crystals as a function of patterns per image were obtained from 100 indexing trials of different simulated images. The FELIX algorithm was found to perform quite differently in each case. Comparing the trends for the number of correctly indexed crystals (blue) as the number of patterns per image was increased to 15, FELIX indexed fewer RHO-G6 crystals than lysozyme and AT
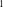
R. However, the fraction of correctly indexed crystals (red) decreased for these lower-symmetry cases, indicating that FELIX found more crystals than are actually in the image. It should be noted that the deviation of this quantity from 1 for AT
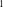
R in the limit of one pattern per image is due to choosing FELIX parameters that optimized its accuracy for up to ten patterns per image. Other parameters were also found that yielded a correctly indexed crystal fraction of 100% for up to three crystals per image, but a worse performance for more.

As the number of crystal patterns per image was increased beyond 15, the slope of the correctly indexed crystal trend is seen to slightly increase for RHO-G6, slightly decrease for lysosyme and plateau for AT
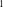
R. Meanwhile, the fraction of correctly indexed crystals remained above 90% for RHO-G6 with up to 45 patterns. For lysozyme this parameter leveled off, while for AT
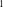
R it was found to drop significantly. These trends with many crystals per pattern confirm that as the crystal symmetry is decreased the accuracy of the FELIX indexing decreases, as expected from an increase of overlapping *hkl* families.

However, the performance with less than 15 crystals per pattern shows that accuracy is not necessarily the whole story, as the number of correctly indexed crystals with a higher symmetry (RHO-G6) was lower than that of lower symmetry (AT
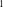
R). Therefore, in practice, a compromise between quantity and quality is necessary when determining the parameters of FELIX. It is worth pointing out that in all cases some patterns were correctly indexed in images containing as many as 50, showing that even in this extreme situation useful information can be extracted. Then, the challenge becomes determining which orientations are indexed correctly. In this direction, some useful metrics that have been found to indicate when the accuracy of the FELIX indexing is poor will be presented in the following experimental study.

### 3.2. Experimental SFX data   

The FELIX algorithm was tested on experimental data collected at the CXI instrument of LCLS from hen egg white lysozyme microcrystals dispersed in a liquid jet. Data from this experiment have been previously used to solve the structure to 1.32 Å (Boutet *et al.*, 2012 ▸), and processed images can be obtained from the coherent X-ray imaging data bank (CXIDB) ID 17 (Boutet, 2013 ▸). For our study, the raw data images from runs 300–320 were reprocessed, sorted into hits and non-hits using the program *Cheetah* (Barty *et al.*, 2014 ▸). A total of 65 046 images were found to be hits, corresponding to 5.7% of the total images that were collected. The unit-cell parameters and the detector geometry were refined using the results of indexing one crystal per frame.

**Figure 4:**
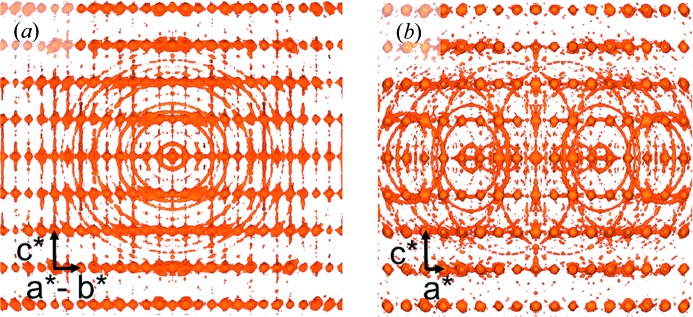
Two views of the merged intensity in three-dimensional reciprocal space: (*a*) along the [110] direction and (*b*) along the [010] direction.

The crystal orientation obtained from this indexing was also used to merge the recorded images into a three-dimensional view of reciprocal space (Yefanov *et al.*, 2014 ▸). The resulting merged reciprocal space shown in Fig. 4 ▸ is found to contain reflections circling the [110] direction of the lysozyme reciprocal lattice. These reflections are assumed to come from multi-crystal images, where only one of the patterns was indexed. Their alignment with respect to the [110] direction suggests that the corresponding crystal agglomerates were stuck together on {110} facets.

Indexing was then performed on the hits using the program *indexamajig* from *CrystFEL* version 0.6.2+6f2696, calling FELIX version 0.31. For a check of data quality, the same images were also indexed with the *MOSFLM* indexer version 7.2.1 (Powell, 1999 ▸), which identified one crystal lattice per image. The spots for each crystal were integrated by *indexa­majig* using the ring method (White *et al.*, 2013 ▸). When multiple crystals were found in an image, the integration of overlapping spots was handled by ignoring pixels that were attributed to the integration region of more than one spot. Furthermore, the background of a spot was estimated by ignoring integration regions from nearby indexed spots. When an integration region or background region contained less than four unmasked pixels, the spot was ignored.

The FELIX parameters were determined by maximizing the number of indexed crystals while minimizing the 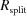 metric obtained from the dataset. The parameters were initially screened by varying 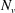, 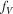 and σ, keeping those that resulted in the highest indexed fraction and number of found crystals. As will be discussed later, trends in the 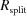 metric were then used to refine the parameters and put further restrictions on the minimum number of crossing geodesics (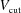) and fraction of spots observed (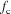) in a crystal pattern. The parameters that resulted in the most indexed crystals with the lowest 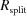 were then 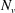 = 150, 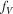 = 0.35, σ = 0.2, 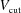 = 30 and 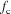 = 0.5.

**Figure 5:**
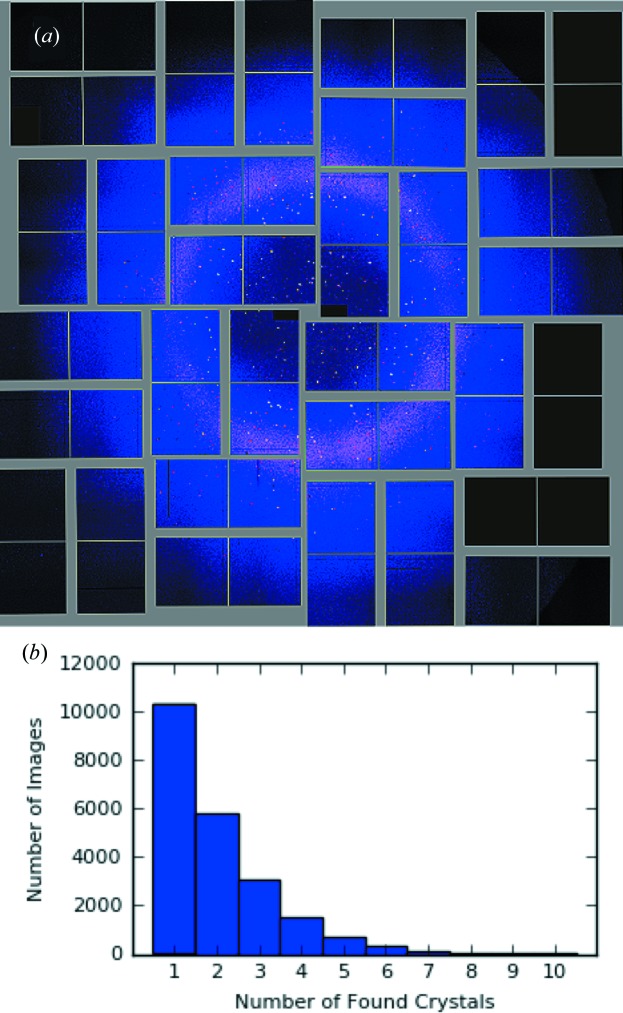
Results of indexing the lysozyme CXIDB data with FELIX. (*a*) A recorded image that was found by FELIX to contain five diffraction patterns. (*b*) The distribution of found crystals per image shows a monotonically decreasing trend up to ten crystals.

A comparison of results obtained using these FELIX parameters and *MOSFLM* is given in Table 2 ▸. The FELIX algorithm indexed a comparable number of images to *MOSFLM* but found two times more crystals. An example image that was found to contain five crystal diffraction patterns is shown in Fig. 5 ▸(*a*). It is seen that the spot density in this image is similar to that from the simulations shown in Fig. 3 ▸(*c*). As shown in Fig. 5 ▸(*b*), most images (10 100) were found to have one crystal pattern, but nearly 50% were found to contain multiple patterns. This is much more than the 2.5% expected assuming Poisson statistics but may be explained by the observation that the crystals were sticking together.

**Figure 6:**
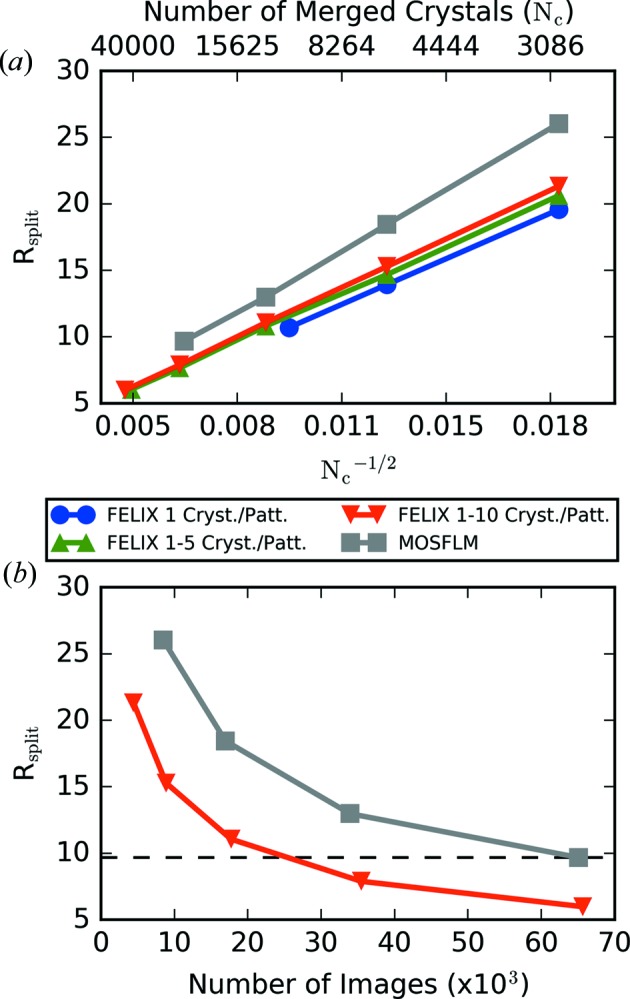
(*a*) The 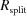 value calculated by merging different subsets of images indexed by FELIX is shown as a function of the number of merged crystals and compared with that obtained from *MOSFLM*. (*b*) The trends of 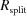 in terms of the number of analyzed images are shown for the final merged FELIX and *MOSFLM* datasets.

The intensities from the indexed patterns were scaled and merged using one iteration of the *partialator* program, without modeling partiality. As already mentioned, the 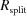 metric (White *et al.*, 2012 ▸) was used to assess the quality of the intensities obtained from multi-crystal images. This quantity was calculated by splitting the images into two subsets, merging the intensities in each subset and computing 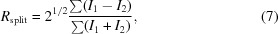
where the sum is carried out over all *hkl* reflections and 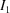 and 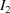 are the merged *hkl* intensites from each subset. The trends of 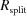 for image subsets with a maximum number of found crystals per image are shown in Fig. 6 ▸(*a*). As expected, these trends decrease with the number of crystals merged (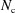) and scale linearly with 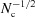. All of the FELIX trends shown in Fig. 6 ▸(*a*) are clustered together and lie under that obtained using *MOSFLM*, suggesting that the indexing results are of a sufficient quality.

When the indexing and integration parameters were not optimum it was found that these trends had a significantly larger slope as the maximum number of crystals per image was increased. By plotting the histograms of integrated intensities for some strong reflections, a direct correlation was found between the amount that the 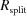 trends sloped upward and the fraction of spots with an integrated intensity near zero. Therefore, often predicting spots where there were none was found to increase 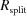. This incorrect prediction was not just due to misindexing; it was also found that the automated procedure in *CrystFEL* for determining the spot profile radius did not perform well with multi-crystal images. To avoid this, a profile radius of 0.0086 nm^−1^, obtained from the one-crystal images, was fixed for both FELIX and *MOSFLM* spot integration.

As shown in Table 2 ▸, the higher number of crystals indexed by FELIX led to an improved signal-to-noise ratio (SNR), 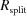 and CC* compared to those found using *MOSFLM*. The trends of 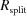 in terms of the number of hit images that were given to the indexer (analyzed images) are shown for both datasets in Fig. 6 ▸(*b*). Plotting these trends in terms of this quantity instead of the number of indexed images considers the different indexed fraction in the two cases. The figure shows that the FELIX trend lies consistently below that of *MOSFLM* as the number of analyzed images is increased. Also, notably, the higher number of indexed crystals in the FELIX dataset translates to needing half the images to achieve the final 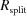 of *MOSFLM*.

**Figure 7:**
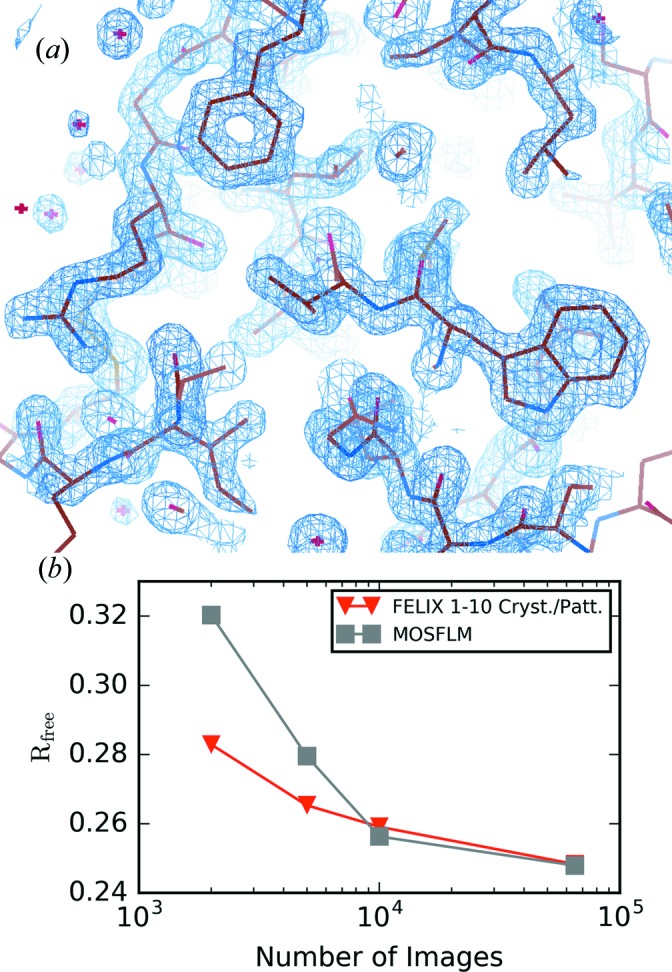
(*a*) The electron density recovered using the dataset indexed by FELIX contoured at 1.5σ (blue) shows good agreement with the protein structure. (*b*) The refined 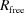 value from FELIX and *MOSFLM* datasets is shown in terms of the number of analyzed images on a log scale.

The merged intensities were then imported into the *Phenix* macromolecular structure solution program (Adams *et al.*, 2010 ▸) and the *phenix.refine* module was used to refine the structure by molecular replacement (Afonine *et al.*, 2012 ▸). PDB entry 1vds (S. Aibara, A. Suzuki, A. Kidera, K. Shibata, T. Yamane, L. J. DeLucas & M. Hirose, in preparation) was used as the initial structure and five refinement cycles were performed in each case. A resolution cutoff of 1.7 Å was imposed for both the *MOSFLM* and the FELIX indexed datasets, corresponding to the resolution where the merged intensity SNR fell below 2. The resulting electron density solved from the FELIX data is shown in Fig. 7 ▸(*a*) and is in good agreement with the structural model for lysozyme, clearly showing the density of benzene rings. Further data on the refinement statistics for the two datasets are given in Table 2 ▸. The 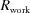 and 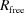 metrics reported here indicate the agreement of the data with the refined atomic model. The similarity of the metrics in the two cases is due to the convergence of this parameter and signifies that the structural information obtained from the FELIX data is on par with that of the *MOSFLM* data. This convergence was studied by performing the same structural refinement with datasets composed of fewer FELIX and *MOSFLM* indexed images. The resulting trends in the 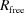 metric in Fig. 7 ▸(*b*) show that it has nearly converged after analyzing just 10 000 hit images for both datasets. Below this point, the 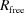 value obtained using FELIX is lower as more crystals were contained in the merge of fewer images. In fact, in this region roughly half as many images are needed for the FELIX dataset to achieve the same 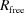, which is consistent with the 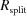 metric behavior shown previously.

Turning our attention to the observation that the crystals were sticking together, analyzing the relative orientations of the crystals found by FELIX allows insight into the microstructure of these agglomerates. The relative orientation is often given in terms of the misorientation angle in grain boundary studies. This is the minimum rotation needed to go from one crystal orientation to another and is analogous to the case depicted in Fig. 1 ▸(*a*). This quantity and the corresponding rotation axis were calculated for all relative orientations between different crystals found in an image, accounting for symmetry-equivalent operations.

**Figure 8:**
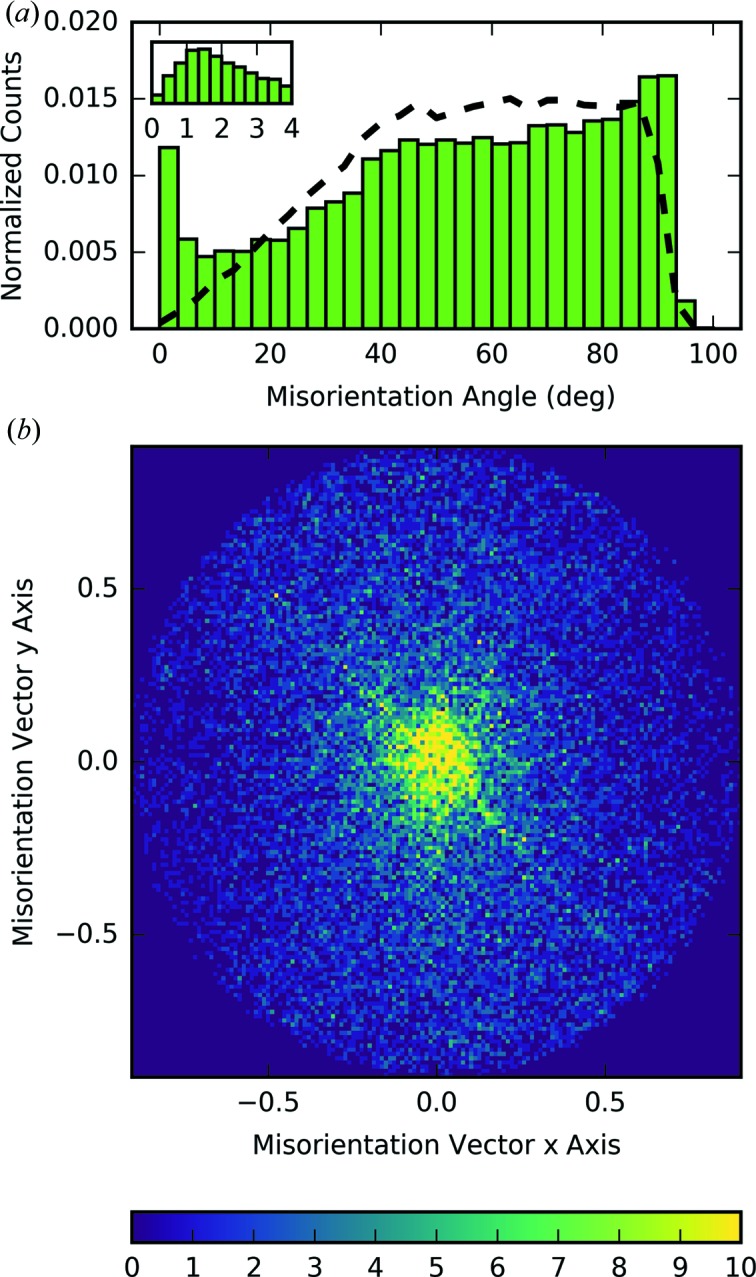
(*a*) The histogram of the misorientation angle between crystals indexed by FELIX (green bars) is compared with the distribution expected for a random system (dashed line). The inset shows a zoomed view of the distribution near 0°. (*b*) The two-dimensional projected density of the FELIX-obtained misorientation vectors is shown. The misorientation vector *x* and *y* axes correspond to the *a* and *b* axes of the lysozyme unit cell.

The distribution of misorientation angles in the FELIX dataset is compared with that which one expects from a random distribution (Morawiec, 1995 ▸) in Fig. 8 ▸(*a*). A larger fraction of angles below 40° with a peak around 1° are found in the experimental data than expected for a random distribution. This is evidence of an abundance of low-angle interfaces in the crystallite agglomerates. The symmetry of these interfaces was investigated by binning the misorientation vectors in three-dimensional RF space, which are determined by the misorientation angle and axis *via* equation (1). The result of projecting these vectors onto the RF *xy* plane is shown in Fig. 8 ▸(*b*). The circularity of the bright spot at the center indicates that the low-angle crystallite boundaries were not found to occur in a preferential direction. For larger misorientation vectors, a diagonal line of higher misorientation vector density along the [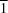
10] direction is clearly seen. This direction agrees with the axis of the powder rings found in the merged three-dimensional intensity of Fig. 4 ▸. The projections of the difference misorientation vector density onto the *yz* and *xz* planes were also examined, and this sharp line was only found to exist in the *xy* plane. Therefore, analysis of the FELIX indexing also found that the lysozyme crystals had a slight tendency to stick together on (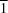
10) facets. It is unclear why a preference for misorientation vectors is not also found along the symmetry-equivalent [110] direction. However, it is not believed to be due to a bias in the indexer as the reflection rings were also seen around only one direction in the three-dimensional merged intensity.

## 4. Discussion   

Spot overlap was handled during integration by discarding overlapping predicted spots in an image. This strategy relies on the correct identification of all of the crystals contributing to an image. Failure to identify a crystal would mean that overlaps could be missed, leading to inaccurate intensity measurements in the dataset. Since a decrease in the data quality was not observed, it is believed that unidentified spot overlap was not prevalent in the analyzed dataset. However, it is expected that this will become more of a problem as the image spot density or number of crystals in an image increases. This might warrant the development of an overlap check during scaling and merging that rejects outliers in integrated spots of the crystal.

As described, multiple attempts at indexing with different FELIX parameters are necessary to optimize the results from a dataset. While this can be cumbersome, an automatic optimization, where a matrix of parameters is tried for a given image, is not currently feasible because of the computation time. Processing an image with FELIX on a single core was found in a few cases to take a few minutes, largely dominated by the ray tracing operations. Then trying sets of different parameters on datasets that contain 100 000 images would require in the worst case more than a year of computation time on a single processor. The FELIX algorithm is planned to be implemented on graphics processing units, which should reduce the computation time enough to enable automatic parameter optimization.

In conclusion, the presented FELIX algorithm is fundamentally different from ‘subtract-and-retry’ methods because its searching of Rodrigues–Frank space is able to disentangle the spots associated with each crystal in snapshot images in a single step. Its performance has been shown to be dependent on the symmetry of the crystal lattice, and the analysis of experimental multiple-crystal images has yielded a dataset with twice as many indexed crystal patterns and improved data quality metrics. As a result, half as many images were necessary to achieve the same data quality as when indexing one crystal per image. This suggests that the data collection time of serial crystallography experiments could be drastically reduced by intentionally collecting multi-crystal images. It could also offer a solution for efficient data collection when the X-ray source repetition rate is faster than the detector readout, as is the case for the proposed 4.5 MHz burst mode of the European XFEL. Details about how to use FELIX in *CrystFEL* are provided in the *CrystFEL* manual and the FELIX binary can be obtained upon request.

## Figures and Tables

**Table 1:** List of crystal parameters, scattering geometry parameters and optimally determined FELIX parameters for the presented simulated structures

	RHO-G6	Lysozyme	AT_1_R
PDB/CIF	nature14575-s3	2lyz	4yay
Space group	*I* 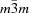	*P* 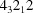	*C*121
Laue class	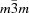	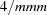	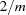
*a*, *b*, *c* (nm)	6.39	7.90, 7.90, 3.80	7.28, 4.10, 16.77
α, β, γ (°)	90.0	90.0	90.0, 99.4, 90.0
*V* (nm^3^)	261.4	237.1	493.8
X-ray energy (eV)	9000	9340	7800
Detector distance (m)	0.090	0.090	0.130
Resolution (Å)	2.0	3.0	3.0
Spots/crystal	220	60	65
*N*_v_	300	400	600
*f*_*V*_	0.7	0.5	0.3
σ_2θ_ = σ_η_ (°)	0.3	0.15	0.15

**Table 2:** Dataset and structure refinement statistics for lysozyme SFX data analyzed by the FELIX and *MOSFLM* indexers

	FELIX	*MOSFLM*
No. images analyzed	65 046	65 046
No. images indexed	21 971	22 917
No. crystals found	44 465	22 917
Resolution range	39.5–1.7	39.5–1.7
*R*_split_ (%)/CC*	5.9/0.99	9.7/0.98
Overall SNR	15.07	9.42
*B*_iso_ (Å^2^)	17.14	15.86
*R*_work_/*R*_free_	0.213/0.248	0.210/0.248
RMSD bonds/angles	0.006/0.81	0.006/0.85
